# The Dynamical Behaviors in a Stochastic SIS Epidemic Model with Nonlinear Incidence

**DOI:** 10.1155/2016/5218163

**Published:** 2016-06-23

**Authors:** Ramziya Rifhat, Qing Ge, Zhidong Teng

**Affiliations:** College of Mathematics and Systems Science, Xinjiang University, Urumqi 830046, China

## Abstract

A stochastic SIS-type epidemic model with general nonlinear incidence and disease-induced mortality is investigated. It is proved that the dynamical behaviors of the model are determined by a certain threshold value ${\tilde{R}}_{0}$. That is, when ${\tilde{R}}_{0}<1$ and together with an additional condition, the disease is extinct with probability one, and when ${\tilde{R}}_{0}>1$, the disease is permanent in the mean in probability, and when there is not disease-related death, the disease oscillates stochastically about a positive number. Furthermore, when ${\tilde{R}}_{0}>1$, the model admits positive recurrence and a unique stationary distribution. Particularly, the effects of the intensities of stochastic perturbation for the dynamical behaviors of the model are discussed in detail, and the dynamical behaviors for the stochastic SIS epidemic model with standard incidence are established. Finally, the numerical simulations are presented to illustrate the proposed open problems.

## 1. Introduction

Our real life is full of randomness and stochasticity. Therefore, using stochastic dynamical models can gain more real benefits. Particularly, stochastic dynamical models can provide us with some additional degrees of realism in comparison to their deterministic counterparts. There are different possible approaches which result in different effects on the epidemic dynamical systems to include random perturbations in the models. In particular, the following three approaches are seen most often. The first one is parameters perturbation; the second one is the environmental noise that is proportional to the variables; and the last one is the robustness of the positive equilibrium of the deterministic models.

In recent years, various types of stochastic epidemic dynamical models are established and investigated widely. The main research subjects include the existence and uniqueness of positive solution with any positive initial value in probability mean, the persistence and extinction of the disease in probability mean, the asymptotical behaviors around the disease-free equilibrium and the endemic equilibrium of the deterministic models, and the existence of the stationary distribution as well as ergodicity. Many important results have been established in many literatures, for example, [1–16] and the references cited therein. Particularly, for stochastic SI type epidemic models, in [6], Gray et al. constructed a stochastic SIS epidemic model with constant population size where the authors not only obtained the existence of the unique global positive solution with any positive initial value, but also established the threshold value conditions; that is, the disease dies out or persists. Furthermore, in the case of the persistence, the authors also showed the existence of a stationary distribution and finally computed the mean value and variance of the stationary distribution.

However, from articles [1–16] and the references cited therein, we see that there are still many important problems which are not studied completely and impactfully. For example, see the following.

1. The stochastic epidemic models with general nonlinear incidence are not investigated. Up to now, only some special cases of nonlinear incidence, for example, saturated incidence rate, are considered. But, we all know that the nonlinear incidence rate in the theory of mathematical epidemiology is very important.
2. For the stochastic epidemic models with the standard incidence, up to now, we do not find any interesting researches.
3. The conditions obtained on the existence of unique stationary distribution are very rigorous. Whether there is a unique stationary distribution only when the model is permanent in the mean with probability one is still an open problem.

Motivated by the above work, in this paper, we consider the following deterministic SIS epidemic model with nonlinear incidence rate and disease-induced mortality:

(1) dStdt=Λ−βfSt,It+γIt−μSt,dItdt=βfSt,It−μ+γ+αIt.

In model (1), *S* and *I* denote the susceptible and infectious individuals, Λ denotes the recruitment rate of the susceptible, *μ* is the natural death rate of *S* and *I*, *α* is the disease-related death rate, the transmission of the infection is governed by a nonlinear incidence rate *βf*(*S*, *I*), where *β* denotes the transmission coefficient between compartments *S* and *I*, *f*(*S*, *I*) is a continuously differentiable function of *S* and *I*, and *γ* denotes the per capita disease contact rate.

Now, we assume that the random effects of the environment make the transmission coefficient *β* of disease in deterministic model (1) generate random disturbance. That is, $\beta \to \beta +\sigma \dot{B}(t)$, where *B*(*t*) is a one-dimensional standard Brownian motion defined on some probability space. Thus, model (1) will become into the following stochastic SIS epidemic model with nonlinear incidence rate:

(2) dSt=Λ−βfSt,It+γIt−μStdt−σfSt,ItdBt,dIt=βfSt,It−μ+γ+αItdt+σfSt,ItdBt.

In this paper, we investigate the dynamical behaviors of model (2). By using the Lyapunov function method, Itô's formula, and the theory of stochastic analysis [17, 18], we will establish a series of new interesting criteria on the extinction of the disease, permanence in the mean of the model with probability one. The stochastic oscillation of the disease about a positive number in the case where there is not disease-related death is also obtained. Further, we study the positive recurrence and the existence of stationary distribution for model (2), and a new criterion is established. Particularly, the effects of the intensities of stochastic perturbation for the dynamical behaviors of the model are discussed in detail. For some special cases of nonlinear incidence *f*(*S*, *I*), for example, *f*(*S*, *I*) = *SI*/*N* (standard incidence) and *f*(*S*, *I*) = *h*(*S*)*g*(*I*), many idiographic criteria on the extinction, permanence, and stationary distribution are established. Lastly, some affirmative answers for the open problems which are proposed in this paper also are given by the numerical examples (the numerical simulation method can be found in [19]).

The organization of this paper is as follows. In Section 2, the preliminaries are given, and some useful lemmas are introduced. In Section 3, the sufficient conditions are established which ensure that the disease dies out with probability one. In Section 4, we establish the sufficient conditions which ensure that the disease in model (2) is permanent in the mean with probability one, and when there is not disease-related death the disease oscillates stochastically about a positive number. In Section 5, the existence on the unique stationary distribution of model (2) is proved. In Section 6, the numerical simulations are carried out to illustrate some open problems. Lastly, a brief discussion is given in the end to conclude this work.

## 2. Preliminaries

Denote *R*_+_^2^ = {(*x*_1_, *x*_2_) : *x*_1_ > 0, *x*_2_ > 0}, *R*_+0_ = [0, *∞*), and *R*_+_ = (0, *∞*). Throughout this paper, we assume that model (2) is defined on a complete probability space (*Ω*, {*F*_*t*_}_*t*≥0_, *P*) with a filtration {*F*_*t*_}_*t*≥0_ satisfying the usual conditions; that is, {*F*_*t*_}_*t*≥0_ is right continuous and *F*_0_ contains all *P*-null sets.

In model (2), *S* and *I* denote the susceptible and infected fractions of the population, respectively, and *N* = *S* + *I* is the total size of the population among whom the disease is spreading; the parameters Λ, *μ*, *β*, and *γ* are given as in model (1); the transmission of the infection is governed by a nonlinear incidence rate *βSg*(*I*); *B*(*t*) denotes one-dimensional standard Brownian motion defined on the above probability space; and *σ* represents the intensity of the Brownian motion *B*(*t*). Throughout this paper, we always assume the following.

(H) *f*(*S*, *I*) is two-order continuously differentiable for any *S* ≥ 0, *I* ≥ 0, and *S* + *I* > 0. For each fixed *I* ≥ 0, *f*(*S*, *I*) is increasing for *S* > 0 and for each fixed *S* ≥ 0, *f*(*S*, *I*)/*I* is decreasing for *I* > 0. *f*(*S*, 0) = *f*(0, *I*) = 0 for any *S* > 0 and *I* > 0, and ∂*f*(*S* ^0^, 0)/∂*I* > 0, where *S* ^0^ = Λ/*μ*.

Particularly, when *f*(*S*, *I*) = *h*(*S*)*g*(*I*), then assumption (**H**) becomes in the following form:

 ($H^{*}$) *h*(*S*) and *g*(*I*) are continuously differentiable for *S* ≥ 0 and *I* ≥ 0, *h*(*S*) is increasing for *S* ≥ 0, and *g*(*I*)/*I* is decreasing for *I* > 0.

**Remark 1 .**

From (**H**), by simple calculating, we can obtain that for any *S* > 0 and *I* > 0, 0 ≤ *f*(*S*, *I*) ≤ (∂*f*(*S*, 0)/∂*I*)*I*, and for any *S*_2_ > *S*_1_ > 0, ∂*f*(*S*_2_, 0)/∂*I* ≥ ∂*f*(*S*_1_, 0)/∂*I*.

**Remark 2 .**

When *f*(*S*, *I*) = *SI*/*N* (standard incidence), where *N* = *S* + *I*, *f*(*S*, *I*) = *SI*/(1 + *ω*_1_ *I* + *ω*_2_ *S*) (Beddington-DeAngelis incidence) with constants *ω*_1_ ≥ 0 and *ω*_2_ ≥ 0, and *f*(*S*, *I*) = *SI*/(1 + *ωI* ^2^) with constant *ω* ≥ 0, then (**H**) is satisfied.

Now, we give the following result for function *f*(*S*, *I*).

**Lemma 3 .**

For any constants *p* > *q* > 0, let *D* = {(*S*, *I*) : *S* > 0, *I* > 0, *q* ≤ *S* + *I* ≤ *p*}. Then,

(3) maxS,I∈DfS,IS,fS,II<∞,

(4) maxS,I∈D1I∂fS,I∂I−fS,II2,1I∂fS,I∂S<∞.

The proof of Lemma 3 is simple. In fact, from (**H**), we have

(5) limS→0fS,IS=∂f0,I∂S,limI→0fS,IS=∂fS,0∂I.

Hence, conclusion (3) holds. Define the functions

(6) HS,I=1I∂fS,I∂I−fS,II2,I>0,12∂2fS,0∂I2,I=0,S,I∈D,GS,I=1I∂fS,I∂I,I>0,∂2fS,0∂I∂S,I=0,S,I∈D.

Using the L'Hospital principle, from (**H**), we have

(7) limI→01I∂fS,I∂I−fS,II2=12∂2fS,0∂I2,limI→01I∂fS,I∂S=∂2fS,0∂I∂S.

This shows that *H*(*S*, *I*) and *G*(*S*, *I*) are continuous for (*S*, *I*) ∈ *D*. Therefore, conclusion (4) also is true.

Next, on the existence of global positive solutions and the ultimate boundedness of solutions for model (2) with probability one, we have the result as follows.

**Lemma 4 .**

For any initial value (*S*(0), *I*(0)) ∈ *R*_+_^2^, model (2) has a unique solution (*S*(*t*), *I*(*t*)) defined on *t* ∈ *R*_+0_ satisfying (*S*(*t*), *I*(*t*)) ∈ *R*_+_^2^ for all *t* ≥ 0 with probability one. Furthermore, when *α* > 0 then *S*_0_ ≤ lim inf_*t*→*∞*_ *N*(*t*) ≤ lim sup_*t*→*∞*_ *N*(*t*) ≤ *S* ^0^, and when *α* = 0 then lim_*t*→*∞*_ *N*(*t*) = *S* ^0^, where *N*(*t*) = *S*(*t*) + *I*(*t*) and *S*_0_ = Λ/(*μ* + *α*).

Lemma 4 can be proved by using the method which is given in [6]. We hence omit it here.

## 3. Extinction of the Disease

Define the constants

(8) R0=β∂fS0,0/∂Iμ+γ+α,R~0=R0−σ2∂fS0,0/∂I22μ+γ+α.

We have that *R*_0_ is the basic reproduction number of deterministic model (1). On the extinction of the disease in probability for model (2) we have the following result.

**Theorem 5 .**

Assume that one of the following conditions holds:

(a) *σ* ^2^ ≤ *β*/(∂*f*(*S* ^0^, 0)/∂*I*) and ${\tilde{R}}_{0}<1$;
(b) *σ* ^2^ > *β* ^2^/2(*μ* + *γ* + *α*).

Then disease *I* in model (2) is extinct with probability one. That is, for any initial value (*S*(0), *I*(0)) ∈ *R*_+_^2^, solution (*S*(*t*), *I*(*t*)) of model (2) has lim_*t*→*∞*_ *I*(*t*) = 0 a.s.

**Proof**

By Lemma 4 we have (*S*(*t*), *I*(*t*)) ∈ *R*_+_^2^ a.s. for all *t* ≥ 0 and lim sup_*t*→*∞*_(*S*(*t*) + *I*(*t*)) ≤ *S* ^0^. For any *η* > 0 there is *T*_0_ > 0 such that *S*(*t*) + *I*(*t*) < *S* ^0^ + *η* for all *t* ≥ *T*_0_. Hence, for any *t* ≥ *T*_0_,

(9) $$\begin{array}{c} \frac{f\left(S\left(t\right),I\left(t\right)\right)}{I\left(t\right)}\in \left(\;0,\frac{\partial f\left(S^{0}+\eta ,0\right)}{\partial I}\;\right]. \end{array}$$

With Itô's formula (see [17, 18]), we have

(10) dlog⁡It=βfSt,ItIt−μ+γ+α−σ22fSt,ItIt2dt+σ·fSt,ItItdBt.

Hence, for any *ε* > 0,

(11) log⁡Itt≤log⁡I0t+β+εt∫0tfSs,IsIsds−μ+γ+α−σ221t∫0tfSs,IsIs2ds+σt∫0tfSs,IsIsdBs.

Define a function

(12) $$\begin{array}{c} g\left(\;u\;\right)=\left(\;\beta +\varepsilon \;\right)u-\frac{\sigma^{2}}{2}u^{2}-\left(\;\mu +\gamma +\alpha \;\right). \end{array}$$

When *σ* = 0, *g*(*u*) is monotone increasing for *u* ∈ *R*_+_, and when *σ* > 0, *g*(*u*) is monotone increasing for *u* ∈ [0, (*β* + *ε*)/*σ* ^2^) and monotone decreasing for *u* ∈ [(*β* + *ε*)/*σ* ^2^, *∞*).

If condition (a) holds, then when *σ* = 0, from (9), we directly have

(13) $$\begin{array}{c} g\left(\;\frac{f\left(S\left(t\right),I\left(t\right)\right)}{I\left(t\right)}\;\right)\leq g\left(\;\frac{\partial f\left(S^{0}+\eta ,0\right)}{\partial I}\;\right)\;\forall t\geq T_{0}. \end{array}$$

When *σ* > 0, since ∂*f*(*S* ^0^, 0)/∂*I* ≤ *β*/*σ* ^2^, we can choose *η* > 0 such that *η* ≤ *ε* and ∂*f*(*S* ^0^ + *η*, 0)/∂*I* < (*β* + *ε*)/*σ* ^2^. From (9) we also have inequality (13). Hence, when *t* ≥ *T*_0_,

(14) log⁡Ittlog⁡I0t+1t∫0tgfSs,IsIsds+σt∫0tfSs,IsIsdBs≤log⁡I0t+1t∫0T0gfSs,IsIsds+1tg∂fS0+η,0∂It−T0+σt∫0tfSs,IsIsdBs.

By the large number theorem for martingales (see [17] or Lemma A.1 given in [9]), we obtain

(15) $$\begin{array}{c} \underset{t\to \infty }{lim\;sup}\frac{\log I\left(t\right)}{t}\leq g\left(\;\frac{\partial f\left(S^{0}+\eta ,0\right)}{\partial I}\;\right)\;\text{a.s.} \end{array}$$

From the arbitrariness of *ε* and *η*, we further obtain

(16) lim supt→∞log⁡Ittβ∂fS0,0∂I−12σ2∂fS0,0∂I2−μ+γ+α=μ+γ+αR~0−1<0a.s.

If condition (b) holds, then, since *σ* > 0, *g*(*u*) has maximum value (*β* + *ε*)^2^/2*σ* ^2^ − (*μ* + *γ* + *α*) at *u* = (*β* + *ε*)/*σ* ^2^, and for any *t* ≥ 0, we have

(17) $$\begin{array}{c} \beta g\left(\;\frac{f\left(S\left(t\right),I\left(t\right)\right)}{I\left(t\right)}\;\right)\leq \frac{{\left(\beta +\varepsilon \right)}^{2}}{2\sigma^{2}}-\left(\;\mu +\gamma +\alpha \;\right), \end{array}$$

which implies

(18) log⁡Itt≤log⁡I0t+β+ε22σ2−μ+γ+α+σt∫0tfSs,IsIsdBs.

With the large number theorem for martingales and arbitrariness of *ε*, we obtain

(19) $$\begin{array}{c} \underset{t\to \infty }{lim\;sup}\frac{\log I\left(t\right)}{t}\leq \frac{\beta^{2}}{2\sigma^{2}}-\left(\;\mu +\gamma +\alpha \;\right)<0\;\text{a.s.} \end{array}$$

From (16) and (19) we finally have lim_*t*→*∞*_ *I*(*t*) = 0 a.s. This completes the proof.

Now, we give a further discussion for conditions (a) and (b) of Theorem 5 by using the intensity *σ* of stochastic perturbation and basic reproduction number *R*_0_ of deterministic model (1).

When *R*_0_ ≤ 1, then, for any *σ* > 0, ${\tilde{R}}_{0}<1$, and it is easy to prove that one of the conditions (a) and (b) of Theorem 5 holds. Therefore, for any *σ* > 0, the conclusions of Theorem 5 hold. Let 1 < *R*_0_ ≤ 2. From ${\tilde{R}}_{0}=1$ we have

(20) $$\begin{array}{c} \sigma ≜\bar{\sigma }=\frac{\sqrt{2\left(\mu +\gamma +\alpha \right)\left(R_{0}-1\right)}}{\partial f\left(S^{0},0\right)/\partial I}. \end{array}$$

Denote

(21) σ1=β2μ+γ+α,σ2=β∂fS0,0/∂I.

Since *σ*_1_ ≤ *σ*_2_, we easily prove that when $\sigma >\bar{\sigma }$ one of the conditions (a) and (b) of Theorem 5 holds. Therefore, for any $\sigma >\bar{\sigma }$, the conclusions of Theorem 5 hold. When *R*_0_ > 2, we have *σ*_1_ > *σ*_2_ and $\sigma_{1}\geq \bar{\sigma }\geq \sigma_{2}$. Hence, condition (a) in Theorem 5 does not hold. We only can obtain that for any *σ* > *σ*_1_ the conclusions of Theorem 5 hold. Summarizing the above discussions we have the following result as a corollary of Theorem 5.

**Corollary 6 .**

Assume that one of the following conditions holds:

(a) *R*_0_ ≤ 1 and *σ* > 0;
(b) 1 < *R*_0_ ≤ 2 and $\sigma >\bar{\sigma }$;
(c) *R*_0_ > 2 and *σ* > *σ*_1_.

Then disease *I* in model (2) is extinct with probability one.

**Corollary 7 .**

Let *f*(*S*, *I*) = *SI*/*N* (standard incidence). Assume that one of the following conditions holds:

(a) *σ* ^2^ ≤ *β* and ${\tilde{R}}_{0}=\beta /(\mu +\gamma +\alpha )-\sigma^{2}/2(\mu +\gamma +\alpha )<1$;
(b) *σ* ^2^ > *β* ^2^/2(*μ* + *γ* + *α*).

Then disease *I* in model (2) is extinct with probability one.

**Corollary 8 .**

Let *f*(*S*, *I*) = *h*(*S*)*g*(*I*). Assume that (**H** ^**∗**^) holds and one of the following conditions holds:

(a) *σ* ^2^ ≤ *β*/*h*(*S* ^0^)*g*′(0) and ${\tilde{R}}_{0}=\beta h\left(S^{0}\right)g^{\prime }(0)/\left(\mu +\gamma +\alpha \right)-\sigma^{2}(h\left(S^{0}\right)g^{\prime }(0){)}^{2}/2(\mu +\gamma +\alpha )<1$;
(b) *σ* ^2^ > *β* ^2^/2(*μ* + *γ* + *α*).

Then disease *I* in model (2) is extinct with probability one.

**Remark 9 .**

It is easy to see that in Theorem 5 the conditions *R*_0_ > 2 and $\bar{\sigma }\leq \sigma \leq \sigma_{1}$ are not included. Therefore, an interesting conjecture for model (2) is proposed; that is, if the above condition holds, then the disease still dies out with probability one. In Section 6, we will give an affirmative answer by using the numerical simulations; see Example 1.

**Remark 10 .**

In the above discussions, we see that case ${\tilde{R}}_{0}=1$ has not been considered. An interesting open problem is whether when ${\tilde{R}}_{0}=1$ the disease in model (2) also is extinct with probability one. A numerical example is given in Section 6; see Example 2.

## 4. Permanence of the Disease

On the permanence of the disease in the mean with probability one for model (2), we establish the following results.

**Theorem 11 .**

If ${\tilde{R}}_{0}>1$, then disease *I* in model (2) is permanent in the mean with probability one. That is, there is a constant *m*_*I*_ > 0 such that, for any initial value (*S*(0), *I*(0)) ∈ *R*_+_^2^, solution (*S*(*t*), *I*(*t*)) of model (2) satisfies

(22) $$\begin{array}{c} \underset{t\to \infty }{lim\;inf}\frac{1}{t}\overset{t}{\underset{0}{\int }}I\left(\;s\;\right)ds\geq m_{I}\;a.s\text{.} \end{array}$$

**Proof**

From ${\tilde{R}}_{0}>1$, we choose a small enough constant *ε* > 0 such that

(23) β∂fS0,0∂I−μ+γ+α−12σ2∂fS0+ε,0∂I2>0.

By Lemma 4, it is clear that, for any initial value (*S*(0), *I*(0)) ∈ *R*_+_^2^, solution (*S*(*t*), *I*(*t*)) of model (2) satisfies lim sup_*t*→*∞*_(1/*t*)∫_0_^*t*^ *I*(*s*)*ds* ≤ *S* ^0^ and for above *ε* > 0 there is *T*_0_ > 0 such that *S*_0_ − *ε* ≤ *S*(*t*) + *I*(*t*) ≤ *S* ^0^ + *ε* a.s. for all *t* ≥ *T*_0_. Denote the set *D*_*ε*_ = {(*S*, *I*) : *S*_0_ − *ε* ≤ *S* + *I* ≤ *S* ^0^ + *ε*}. Since *dN*(*t*) = (Λ − *μN*(*t*) − *αI*(*t*))*dt*, we obtain for any *t* > *T*_0_

(24) ∫T0tSs−S0ds=−μ+αμ∫T0tIsds+NT0−Ntμ.

From (10), for any *t* ≥ *T*_0_,

(25) log⁡It=log⁡I0+β∫0t∂fS0,0∂I+fSs,IsIs−∂fS0,0∂Ids−μ+γ+αt−12σ2∫0tfSs,IsIs2ds+σ∫0tfSs,IsIsdBs.

Since *f*(*S*, *I*)/*I* for *S* > 0 and *I* > 0 is continuously differentiable, lim_*I*→0_(*f*(*S*, *I*)/*I*) = ∂*f*(*S*, 0)/∂*I* exists for any *S* > 0, and set *D*_*ε*_ is convex and connected, by the Lagrange mean value theorem when *t* ≥ *T*_0_ we have

(26) fSt,ItIt−∂fS0,0∂I=1ϕt∂fξt,ϕt∂I−fξt,ϕtϕ2tIt+1ϕt∂fξt,ϕt∂SSt−S0,

where (*ξ*(*t*), *ϕ*(*t*)) ∈ *D*_*ε*_. Let constants

(27) M1=maxS,I∈Dε1I∂fS,I∂I−fS,II2,M2=maxS,I∈Dε1I∂fS,I∂S.

From Lemma 3 we have 0 < *M*_1_, *M*_2_ < *∞*. For any *t* ≥ *T*_0_, we have

(28) 1ϕt∂fξt,ϕt∂I−fξt,ϕtϕ2t≥−M1a.s.,1ϕt∂fξt,ϕt∂S≤M2a.s.

From (25) and Remark 1 we further have

(29) log⁡It=log⁡I0+β∫0T0fSs,IsIsds+β∂fS0,0∂It−T0+β∫T0t1ϕt∂fξt,ϕt∂I−fξt,ϕtϕ2tIs+1ϕt·∂fξt,ϕt∂SSs−S0ds−μ+γ+αt−12σ2∫0tfSs,IsIs2dt+σ∫0tfSs,IsIsdBs≥log⁡I0+β∫0T0fSs,IsIsds+β∂fS0,0∂It−T0−βM1∫T0tIsds+βM2∫T0tSs−S0ds−μ+γ+αt−12σ2∂fS0+ε,0∂I2t+σ∫0tfSs,IsIsdBs=log⁡I0+β∫0T0fSs,IsIsdt+β∂fS0,0∂It−T0−βM1∫T0tIsds−βM2μ+αμ∫T0tIsds+βM21μNT0−Nt−μ+γ+αt−12·σ2∂fS0+ε,0∂I2t+σ∫0tfSs,IsIsdBs=Ht+θt−θ0∫0tSsds,

where

(30) Ht=log⁡I0+β∫0T0fSs,IsIsds−β∂fS0,0∂IT0+βM1+M2μ+αμ∫0T0Isds+βM21μNT0−Nt+σ∫0tfSt,IsIsdBs,θ=β∂fS0,0∂I−μ+γ+α−12σ2∂fS0+ε,0∂I2,θ0=βM1+M2μ+αμ.

By the large number theorem for martingales and Lemma 4, lim_*t*→*∞*_(*H*(*t*)/*t*) = 0 a.s. Therefore, from Lemma  5.2 given in [16], we finally obtain lim inf_*t*→*∞*_(1/*t*)∫_0_^*t*^ *I*(*s*)*ds* ≥ *θ*/*θ*_0_ a.s. This completes the proof.

**Remark 12 .**

From (20), we have that ${\tilde{R}}_{0}>1$ is equivalent to $\sigma <\bar{\sigma }$. Therefore, Theorem 11 also can be rewritten by using intensity *σ* of stochastic perturbation in the following form: if $\sigma <\bar{\sigma }$, then disease *I* in model (2) is permanent in the mean with probability one.

**Remark 13 .**

Combining Corollary 6 and Remark 12 we can obtain that when 1 < *R*_0_ ≤ 2, number $\bar{\sigma }$ is a threshold value. When $0<\sigma <\bar{\sigma }$, the disease *I* in model (2) is permanent in the mean and when $\sigma >\bar{\sigma }$, the disease *I* is extinct with probability one. However, when *R*_0_ > 2, then the alike results are not established. Therefore, it yet is an interesting open problem.

**Theorem 14 .**

Susceptible *S* in model (2) also is permanent in the mean with probability one. That is, there is a constant *m*_*S*_ > 0 such that, for any initial value (*S*(0), *I*(0)) ∈ *R*_+_^2^, solution (*S*(*t*), *I*(*t*)) of model (2) satisfies

(31) $$\begin{array}{c} \underset{t\to \infty }{lim\;inf}\frac{1}{t}\overset{t}{\underset{0}{\int }}S\left(\;s\;\right)ds\geq m_{S}\;a.s\text{.} \end{array}$$

**Proof**

By Lemma 4 we easily see that, for any initial value (*S*(0), *I*(0)) ∈ *R*_+_^2^, solution (*S*(*t*), *I*(*t*)) of model (2) satisfies lim sup_*t*→*∞*_(1/*t*)∫_0_^*t*^ *S*(*s*)*ds* ≤ *S* ^0^ and for any small enough constant *ε* > 0 there is *T*_0_ > 0 such that *S*_0_ − *ε* ≤ *S*(*t*) + *I*(*t*) ≤ *S* ^0^ + *ε* for all *t* ≥ *T*_0_. Hence, by Lemma 3, when *t* ≥ *T*_0_ we have *f*(*S*(*t*), *I*(*t*)) ≤ *M*_*S*_ *S*(*t*), where *M*_*S*_ = max_*D*_*ε*__{*f*(*S*, *I*)/*S*} < *∞*. Integrating the first equation of model (2) we obtain for any *t* ≥ *T*_0_

(32) St−S0t=Λ−1t∫0tβfSs,Is+μSs−γIsds−σt∫0tfSs,IsdBs≥Λ−1t∫0T0βfSs,Is+μSsds−1t∫T0tβMS+μSsds−σt∫0tfSs,IsdBs.

Therefore, with the large number theorem for martingales, we finally have

(33) $$\begin{array}{c} \underset{t\to \infty }{lim\;inf}\frac{1}{t}\overset{t}{\underset{0}{\int }}S\left(\;s\;\right)ds\geq \frac{\Lambda }{\beta M_{S}+\mu }. \end{array}$$

This completes the proof.

As consequences of Theorems 11 and 14, we have the following corollaries.

**Corollary 15 .**

Let *f*(*S*, *I*) = *SI*/*N* (standard incidence). If ${\tilde{R}}_{0}=(\beta -\left(1/2\right)\sigma^{2})/(\mu +\gamma +\alpha )>1$, then model (2) is permanent in the mean with probability one.

**Corollary 16 .**

Let *f*(*S*, *I*) = *h*(*S*)*g*(*I*). Assume that (**H** ^**∗**^) holds and ${\tilde{R}}_{0}=\beta h\left(S^{0}\right)g^{\prime }(0)/\left(\mu +\gamma +\alpha \right)-\sigma^{2}{\left(h\left(S^{0}\right)g^{\prime }\left(0\right)\right)}^{2}/2(\mu +\gamma +\alpha )>1$; then model (2) is permanent in the mean with probability one.

We further have the result on the weak permanence of model (2) in probability.

**Corollary 17 .**

Assume that ${\tilde{R}}_{0}>1$. Then there is a constant *ξ* > 0 such that, for any initial value (*S*(0), *I*(0)) ∈ *R*_+_^2^, solution (*S*(*t*), *I*(*t*)) of model (2) satisfies

(34) lim supt→∞ It≥ξ,lim supt→∞ St≥ξa.s.

Now, we discuss special case: *α* = 0 for model (2); that is, there is not disease-related death in model (2). We can establish the following more precise results on the weak permanence of the disease in probability compared to the conclusion given in Corollary 17.

**Theorem 18 .**

Let *α* = 0 in model (2). If ${\tilde{R}}_{0}>1$, then, for any initial value (*S*(0), *I*(0)) ∈ *R*_+_^2^, solution (*S*(*t*), *I*(*t*)) of model (2) satisfies

(35) lim supt→∞ It≥ξa.s.,

(36) lim inft→∞ It≤ξa.s.,

where *ξ* > 0 satisfies the equation

(37) $$\begin{array}{c} \frac{f\left(S^{0}-\xi ,\xi \right)}{\xi }=\left\{\begin{array}{cc} \frac{\mu +\gamma }{\beta }, & \sigma =0,\\ \frac{2\left(\mu +\gamma \right)}{\beta +\sqrt{\beta^{2}-2\sigma^{2}\left(\mu +\gamma \right)}}, & \sigma >0. \end{array}\right. \end{array}$$

**Proof**

From Lemma 4, we know that lim_*t*→*∞*_(*S*(*t*) + *I*(*t*)) = *S* ^0^. Without loss of generality, we assume that *S*(*t*) + *I*(*t*) ≡ *S* ^0^ for all *t* ≥ 0. From (10), for any *t* ≥ 0,

(38) log⁡It=log⁡I0+∫0tβfS0−Is,IsIs−μ+γ−σ22fS0−Is,IsIs2ds+∫0tσfSt,IsIsdBs.

Define a function *u*(*I*) = *f*(*S* ^0^ − *I*, *I*)/*I*. Then, for any *t* ≥ 0,

(39) log⁡It=log⁡I0+∫0tguIsds+∫0tσfSs,IsIsdBs,

where function *g*(*u*) = *βu* − (*σ* ^2^/2)*u* ^2^ − (*μ* + *γ*). With condition ${\tilde{R}}_{0}>1$ we have *g*(0) = −(*μ* + *γ*) < 0 and

(40) g∂fS0,0∂I−σ22∂fS0,0∂I2+β∂fS0,0∂I−μ+γ>0.

Hence, *g*(*u*) = 0 has a positive root *η* in (0, ∂*f*(*S* ^0^, 0)/∂*I*) which is

(41) $$\begin{array}{c} \eta =\left\{\begin{array}{cc} \frac{\mu +\gamma }{\beta }, & \sigma =0,\\ \frac{2\left(\mu +\gamma \right)}{\beta +\sqrt{\beta^{2}-2\sigma^{2}\left(\mu +\gamma \right)}}, & \sigma >0. \end{array}\right. \end{array}$$

Since *u*(*I*) is monotone decreasing for *I* ∈ (0, *S* ^0^), *u*(*S* ^0^) = 0, and

(42) $$\begin{array}{c} \underset{I\to 0^{+}}{lim}u\left(\;I\;\right)=\underset{I\to 0^{+}}{lim}\frac{f\left(S^{0}-I,I\right)}{I}=\frac{\partial f\left(S^{0},0\right)}{\partial I}, \end{array}$$

there is a unique *ξ* ∈ (0, *S* ^0^) such that *u*(*ξ*) = *f*(*S* ^0^ − *ξ*, *ξ*)/*ξ* = *η* and *g*(*u*(*ξ*)) = *g*(*η*) = 0.

When *σ* > 0 and *β*/*σ* ^2^ < ∂*f*(*S* ^0^, 0)/∂*I*, since function *g*(*u*) has maximum value *g*(*β*/*σ* ^2^) at *u* = *β*/*σ* ^2^ and *g*(*β*/*σ* ^2^) > *g*(∂*f*(*S* ^0^, 0)/∂*I*), there is a unique $\hat{I}$, such that $u(\hat{I})=\beta /\sigma^{2}$. From *η* ∈ (0, ∂*f*(*S* ^0^, 0)/∂*I*) and *g*(*η*) = 0 we have *η* < *β*/*σ* ^2^. Hence, $0<\hat{I}<\xi <S^{0}$.

From the above discussion we obtain that *g*(*u*(*I*)) > 0 is strictly increasing on $I\in (0,\hat{I})$, *g*(*u*(*I*)) > 0 is strictly decreasing on $I\in (\hat{I},\xi )$, and *g*(*u*(*I*)) < 0 is strictly decreasing on *I* ∈ (*ξ*, *S* ^0^).

When *σ* ^2^ ≤ *β*/(∂*f*(*S* ^0^, 0)/∂*I*), similarly to the above discussion, we can obtain that *g*(*u*(*I*)) > 0 is strictly decreasing on *I* ∈ (0, *ξ*) and *g*(*u*(*I*)) < 0 is strictly decreasing on *I* ∈ (*ξ*, *S* ^0^).

Now, we firstly prove that (35) is true. If it is not true, then there is an enough small *ε*_0_ ∈ (0,1) such that *P*(*Ω*_1_) > *ε*_0_, where *Ω*_1_ = {lim sup_*t*→*∞*_ *I*(*t*) < *ξ*}. Hence, for every *ω* ∈ *Ω*_1_, there is a constant *T*_1_ = *T*_1_(*ω*) ≥ *T*_0_ such that

(43) $$\begin{array}{c} I\left(\;t\;\right)\leq \xi -\varepsilon_{0}\;\forall t\geq T_{1}. \end{array}$$

With the above discussion we know that *g*(*u*(*I*(*t*))) ≥ *g*(*u*(*ξ* − *ε*_0_)) > 0 for all *t* ≥ *T*_1_. From (39) we further obtain for any *t* ≥ *T*_1_

(44) log⁡It≥log⁡I0+∫0T1guIsds+guξ−ε0t−T1+∫0tσfSs,IsIsdBs.

From the large number theorem for martingales, we have lim inf_*t*→*∞*_(log⁡*I*(*t*)/*t*) ≤ *g*(*u*(*ξ* − *ε*_0_)) > 0, which implies *I*(*t*) → *∞* as *t* → *∞*. This leads to a contradiction with (43).

Next, we prove that (36) holds. If it is not true, then there is an enough small *ε*_1_ ∈ (0,1) such that *P*(*Ω*_2_) > *ε*_1_, where *Ω*_2_ = {lim inf_*t*→*∞*_ *I*(*t*) > *ξ*}. Hence, for every *ω* ∈ *Ω*_2_, there is *T*_2_ = *T*_2_(*ω*) ≥ *T*_0_ such that

(45) $$\begin{array}{c} I\left(\;t\;\right)\geq \xi +\varepsilon_{1}\;\forall t\geq T_{2}. \end{array}$$

With the above discussion we have *g*(*u*(*I*(*t*))) ≤ *g*(*u*(*ξ* + *ε*_1_)) < 0 for all *t* ≥ *T*_2_. Together with (39), we further obtain for any *t* ≥ *T*_2_

(46) log⁡Itlog⁡I0+∫0T2guIsds+∫T2tguIsds+∫0tσfSs,IsIsdBs≤log⁡I0+∫0T2guIsds+guξ+ε1t−T2+∫0tσfSs,IsIsdBs.

With the large number theorem for martingales, we have lim sup_*t*→*∞*_(log⁡*I*(*t*)/*t*) ≤ *g*(*u*(*ξ* + *ε*_1_)) < 0, which implies *I*(*t*) → 0 as *t* → *∞*. This leads to a contradiction with (45). This completes the proof.

**Remark 19 .**

Theorem 18 indicates that if ${\tilde{R}}_{0}>1$ and *α* = 0, then any solution (*S*(*t*), *I*(*t*)) of model (2) with initial value (*S*(0), *I*(0)) ∈ *R*_+_^2^ oscillates about a positive number *ξ*. Therefore, an interesting open problem is whether there is a more less positive *m* than number *ξ* such that any solution (*S*(*t*), *I*(*t*)) of model (2) with initial value (*S*(0), *I*(0)) ∈ *R*_+_^2^, lim inf_*t*→*∞*_ *I*(*t*) ≥ *m* a.s. In Section 6, we will give an affirmative answer by using the numerical simulations; see Example 3.

From Theorem 18, we easily see that number *ξ* will arise from the change when the noise intensity *σ* changes. Therefore, it is very interesting and important to discuss how number *ξ* changes along with the change of *σ*. We have the following result.

**Theorem 20 .**

Assume that *α* = 0 in model (2) and ${\tilde{R}}_{0}>1$. Let number *ξ* be given in Theorem 18 and *R*_0_ = *β*(∂*f*(*S* ^0^, 0)/∂*I*)/(*μ* + *γ*). Then one has the following.

(a) *ξ* as the function of *σ* is defined for
  (47) $$\begin{array}{c} 0<\sigma <\frac{\sqrt{2\left(\mu +\gamma \right)\left(R_{0}-1\right)}}{\partial f\left(S^{0},0\right)/\partial I}≔\hat{\sigma }. \end{array}$$
(b) *ξ* is monotone decreasing for $\sigma \in (0,\hat{\sigma })$.
(c) lim_*σ*→0_ *ξ* = *I* ^*∗*^, where (*S* ^*∗*^, *I* ^*∗*^) is the endemic equilibrium of deterministic model (1).
(d) If 1 ≤ *R*_0_ ≤ 2, then ${lim}_{\sigma \to \hat{\sigma }}\xi =0$, and if *R*_0_ > 2, then ${lim}_{\sigma \to \hat{\sigma }}\xi =\xi_{2}$, where *ξ*_2_ satisfies
  (48) $$\begin{array}{c} \frac{f\left(S^{0}-\xi_{2},\xi_{2}\right)}{\xi_{2}}=\frac{\partial f\left(S^{0},0\right)/\partial I}{R_{0}-1}. \end{array}$$

**Proof**

Since

(49) $$\begin{array}{c} \frac{f\left(S^{0}-\xi ,\xi \right)}{\xi }=\eta , \end{array}$$

by the inverse function theorem we obtain that *ξ* as the function of *η* is defined for *η* ∈ (0, ∂*f*(*S* ^0^, 0)/∂*I*). From

(50) $$\begin{array}{c} \eta =\frac{\beta -\sqrt{\beta^{2}-2\sigma^{2}\left(\mu +\gamma \right)}}{\sigma^{2}}, \end{array}$$

we can obtain that *η* ∈ (0, ∂*f*(*S* ^0^, 0)/∂*I*) when $0<\sigma <\hat{\sigma }$. Therefore, *ξ* as a function of *σ* is defined for $0<\sigma <\hat{\sigma }$.

Computing the derivative of *η* with respect to *σ*, we have

(51) dηdσ−2βσ3+2μ+γσβ2−2σ2μ+γ+2β2−2σ2μ+γσ3=2β2−2σ2μ+γ−2ββ2−2σ2μ+γσ3β2−2σ2μ+γ.

Since

(52) 2β2−2σ2μ+γ2−2ββ2−2σ2μ+γ2=4σ4μ+γ2>0,

we have *dη*/*dσ* > 0. From the definition of *ξ*, we easily see that *ξ* is monotone decreasing for *η*. From (49) and (**H**), we obtain that *dξ*/*dη* exists and is continuous for *η*. Since (∂/∂*ξ*)(*f*(*S* ^0^ − *ξ*, *ξ*)/*ξ*) < 0, we have *dξ*/*dη* < 0. Therefore, *dξ*/*dσ* = (*dξ*/*dη*)(*dη*/*dσ*) < 0. It follows that *ξ* is monotone decreasing as *σ* increases. Thus, both lim_*σ*→0_ *ξ* and ${lim}_{\sigma \to \hat{\sigma }}\xi$ exist. Let lim_*σ*→0_ *ξ* = *ξ*_1_ and ${lim}_{\sigma \to \hat{\sigma }}\xi =\xi_{2}$. We have

(53) $$\begin{array}{c} \underset{\sigma \to 0}{lim}\;\eta =\underset{\sigma \to 0}{lim}\frac{2\left(\mu +\gamma \right)}{\beta +\sqrt{\beta^{2}-2\sigma^{2}\left(\mu +\gamma \right)}}=\frac{\mu +\gamma }{\beta }. \end{array}$$

Hence, lim_*σ*→0_(*f*(*S* ^0^ − *ξ*, *ξ*)/*ξ*) = lim_*σ*→0_ *η* = (*μ* + *γ*)/*β*. This shows that *f*(*S* ^0^ − *ξ*_1_, *ξ*_1_)/*ξ*_1_ = (*μ* + *γ*)/*β*. Let (*S* ^*∗*^, *I* ^*∗*^) be the endemic equilibrium of deterministic model (1); then we have *f*(*S* ^0^ − *I* ^*∗*^, *I* ^*∗*^)/*I* ^*∗*^ = (*μ* + *γ*)/*β*. Hence, *ξ*_1_ = *I* ^*∗*^. This shows that lim_*σ*→0_ *ξ* = *I* ^*∗*^.

On the other hand, we have

(54) $$\begin{array}{c} \underset{\sigma \to \hat{\sigma }}{lim}\;\eta =\frac{\beta -\sqrt{\beta^{2}-2{\hat{\sigma }}^{2}\left(\mu +\gamma \right)}}{{\hat{\sigma }}^{2}}=\frac{\left(\partial f\left(S^{0},0\right)/\partial I\right)\left(\beta \left(\partial f\left(S^{0},0\right)/\partial I\right)-\left|\beta \left(\partial f\left(S^{0},0\right)/\partial I\right)-2\left(\mu +\gamma \right)\right|\right)}{2\left(\beta \left(\partial f\left(S^{0},0\right)/\partial I\right)-\left(\mu +\gamma \right)\right)}. \end{array}$$

If 1 ≤ *R*_0_ ≤ 2, then from (54) we obtain ${lim}_{\sigma \to \hat{\sigma }}\eta =\partial f(S^{0},0)/\partial I$. Hence,

(55) $$\begin{array}{c} \underset{\sigma \to \hat{\sigma }}{lim}\frac{f\left(S^{0}-\xi ,\xi \right)}{\xi }=\frac{\partial f\left(S^{0},0\right)}{\partial I}. \end{array}$$

This shows that ${lim}_{\sigma \to \hat{\sigma }}\xi =0$. If *R*_0_ > 2, then we have from (54)

(56) limσ→σ^ η∂fS0,0/∂Iμ+γβ∂fS0,0/∂I−μ+γ=∂fS0,0/∂IR0−1,

which implies

(57) $$\begin{array}{c} \underset{\sigma \to \hat{\sigma }}{lim}\frac{f\left(S^{0}-\xi ,\xi \right)}{\xi }=\frac{\partial f\left(S^{0},0\right)/\partial I}{R_{0}-1}. \end{array}$$

Therefore, we have ${lim}_{\sigma \to \hat{\sigma }}\xi =\xi_{2}$, and *ξ*_2_ satisfies

(58) $$\begin{array}{c} \frac{f\left(S^{0}-\xi_{2},\xi_{2}\right)}{\xi_{2}}=\frac{\partial f\left(S^{0},0\right)/\partial I}{\left(R_{0}-1\right)}. \end{array}$$

This completes the proof.

Conclusion (b) of Theorem 20 shows that when *α* = 0 in model (2), number *ξ* monotonically decreases when *σ* increases in $\left(0,\hat{\sigma }\right)$, and when *σ* = 0, *ξ* has a maximum value *I* ^*∗*^ by Conclusion (c). Therefore, 0 < *ξ* < *I* ^*∗*^ when *σ* > 0. If 1 ≤ *R*_0_ ≤ 2, then when $\sigma =\hat{\sigma }$, *ξ* has a minimum value 0 and if *R*_0_ > 2 then when $\sigma =\hat{\sigma }$, *ξ* has a minimum value *ξ*_2_ > 0 by Conclusion (d).

It is clear that when in model (2) *α* = 0 then $\hat{\sigma }=\bar{\sigma }$ from (20). On the other hand, from Conclusion (c) of Corollary 7, we see that if *R*_0_ > 2 then when *σ* > *σ*_1_, where *σ*_1_ is given in (21), we have lim_*t*→*∞*_ *I*(*t*) = 0 a.s. for any solution (*S*(*t*), *I*(*t*)) of model (2) with initial value (*S*(0), *I*(0)) ∈ *R*_+_^2^, which implies that *ξ* = 0. Therefore, when *R*_0_ > 2, we can propose an interesting open problem: whether there is a critical value $\sigma^{*}\in (\hat{\sigma },\sigma_{1})$ such that when *σ* ∈ (0, *σ* ^*∗*^) we have the fact that *ξ* is monotonically decreasing and *ξ* > 0 and when *σ* > *σ* ^*∗*^ we have *ξ* = 0.

**Remark 21 .**

When *R*_0_ > 2, then from (56) we obtain

(59) $$\begin{array}{c} \underset{\sigma \to \hat{\sigma }}{lim}\;\eta =\frac{\left(\partial f\left(S^{0},0\right)/\partial I\right)\left(\mu +\gamma \right)}{\beta \left(\partial f\left(S^{0},0\right)/\partial I\right)-\left(\mu +\gamma \right)}>\frac{\mu +\gamma }{\beta }; \end{array}$$

namely,

(60) limσ→σ^fS0−ξ,ξξ∂fS0,0/∂Iμ+γβ∂fS0,0/∂I−μ+γ>μ+γβ=fS0−I∗,I∗I∗,

where (*S* ^*∗*^, *I* ^*∗*^) is the endemic equilibrium of deterministic model (1). Hence,

(61) $$\begin{array}{c} \frac{f\left(S^{0}-\xi_{2},\xi_{2}\right)}{\xi_{2}}>\frac{f\left(S^{0}-I^{*},I^{*}\right)}{I^{*}}. \end{array}$$

Consequently, 0 < *ξ*_2_ < *I* ^*∗*^.

**Remark 22 .**

When *f*(*S*, *I*) = *SI*, we easily validate that Theorems 20 and 24 degenerate into Theorems  5.1 and 5.4 which are given in [19], respectively. Therefore, Theorems 18 and 20 are the considerable extension of Theorems  5.1 and 5.4 in general nonlinear incidence cases, respectively.

**Remark 23 .**

For the case *α* > 0 in model (2), an interesting and important open problem is when ${\tilde{R}}_{0}>1$ whether we also can establish similar results as Theorems 18 and 20. Furthermore, as an improvement of the results obtained in Corollary 17 we also propose another open problem: only when ${\tilde{R}}_{0}>1$ we also can establish the permanence of the disease with probability one; that is, there is a constant *m* > 0 such that, for any solution (*S*(*t*), *I*(*t*)) of model (2) with initial value (*S*(0), *I*(0)) ∈ *R*_+_^2^, one has lim_*t*→*∞*_ *I*(*t*) ≥ *m*, a.s. In Section 6, we will give an affirmative answer by using the numerical simulations; see Example 3.

## 5. Stationary Distribution

From Theorems 11 and 14 we obtain that when ${\tilde{R}}_{0}>1$ model (2) is permanent in the mean with probability one. However, when ${\tilde{R}}_{0}>1$ model (2) also has a stationary distribution. We have an affirmative answer as follows.

**Theorem 24 .**

If ${\tilde{R}}_{0}>1$, then model (2) is positive recurrent and has a unique stationary distribution.

**Proof**

Here, the method given in the proof of Theorem  5.1 in [17] is improved and developed. By Lemma 4 and Remark 9 we only need to give the proof in region Γ, where Γ = {(*S*, *I*) : *S* ≥ 0, *I* ≥ 0, *S*_0_ ≤ *S* + *I* ≤ *S* ^0^}. Let (*S*(*t*), *I*(*t*)) be any solution of model (1) with (*S*(0), *I*(0)) ∈ Γ a.s. for all *t* ≥ 0. Let *a* > 0 be a large enough constant, and let

(62) $$\begin{array}{c} D=\left\{\;\left(\;S,I\;\right)\in \Gamma :\frac{1}{a}<S<S^{0}-\frac{1}{a},\;\;\frac{1}{a}<I<S^{0}-\frac{1}{a}\;\right\}. \end{array}$$

When (*S*, *I*) ∈ Γ∖*D*, then either 0 < *S* < 1/*a* or 0 < *I* < 1/*a*. The diffusion matrix for model (56) is

(63) $$\begin{array}{c} A\left(\;S,I\;\right)=\left(\begin{array}{cc} \sigma^{2}f^{2}\left(S,I\right) & -\sigma^{2}f^{2}\left(S,I\right)\\ -\sigma^{2}f^{2}\left(S,I\right) & \sigma^{2}f^{2}\left(S,I\right) \end{array}\right). \end{array}$$

For any $(S,I)\in \bar{D}$ we have *σ* ^2^ *f* ^2^(*S*, *I*) ≥ *σ* ^2^(*f*(1/*a*, *S* ^0^ − 1/*a*)/(*aS* ^0^ − 1))^2^.

Choose a Lyapunov function as follows:

(64) $$\begin{array}{c} V\left(\;S,I\;\right)=\Psi_{1}\left(\;I\;\right)+\Psi_{2}\left(\;S,I\;\right)+\Psi_{3}\left(\;S\;\right), \end{array}$$

where

(65) Ψ1I=1vI−v,Ψ2S,I=1vI−vS0−S,Ψ3S=1S,

and 0 < *v* < 1 is a constant. Computing *L*Ψ_1_, by Remark 1, we have

(66) LΨ1=−I−v+1βfS,I−μ+α+γI+121+v·σ2I−v+2f2S,I≤I−vμ+α+γ+121+vσ2∂fS0,0∂I2−β∂fS0,0∂I+I−vβ∂fS0,0∂I−fS,II.

Applying the Lagrange mean value theorem, we have

(67) ∂fS0,0∂I−fS,II1ϕ∂fξ,ϕ∂SS0−S+fξ,ϕϕ2−1ϕ∂fξ,ϕ∂II≤M1S0−S+M2I+M3R,

where (*ξ*, *ϕ*) ∈ Γ and

(68) M1=maxS,I∈Γ1I∂fS,I∂S,M2=maxS,I∈ΓfS,II2−1I∂fS,I∂I.

By Lemma 3, we have 0 ≤ *M*_1_; *M*_2_ < *∞*. We hence have

(69) LΨ1≤I−vμ+α+γ+121+vσ2∂fS0,0∂I2−β∂fS0,0∂I+βM1S0−SI−v+βM2I1−v.

Computing *L*Ψ_2_, by Remark 1, we have

(70) LΨ2=−1vI−vΛ−μS−βfS,I+γI−I−v+1S0−SβfS,I−μ+α+γI+121+v·σ2f2S,II−v+2S0−S−12I−v+1σ2f2S,I=−1vI−vμS0−S−βfS,I+γI−I−vS0−SβfS,II−μ+α+γ+121+v·σ2fS,II2I−vS0−S−σ2fS,II2I1−v=I−vS0−S−μv+μ+α+γ−βfS,II+121+vσ2fS,II2+I1−vβvfS,II−12σ2fS,II2−δvI−v+1≤I−vS0−S·−μv+μ+α+γ+121+vσ2∂fS0,0∂I2+βv∂fS0,0∂I·I1−v−12σ2fS,II2I1−v−δvI−v+1.

Computing *L*Ψ_3_, we have

(71) LΨ3−1S2Λ−μS−βfS,I+γI+1S3σ2f2S,I≤−ΛS2+μS+βfS,IS1S+σ2fS,IS21S−γS2I≤−ΛS2+1Sμ+βM0+σ2M02−γS2I≤−Λ2S2+12Λμ+βM0+σ2M022−γS2I,

where, by Lemma 3, *M*_0_ = max_Γ_{*f*(*S*, *I*)/*S*} < *∞*. From the above calculations, we obtain that for any (*S*, *I*) ∈ Γ∖*D*

(72) LV≤I−vμ+α+γ+121+vσ2∂fS0,0∂I2−β∂fS0,0∂I+I−vS0−S−μv+μ+α+γ+121+vσ2∂fS0,0∂I2+βM1+S01−v·βM2+βv∂fS0,0∂I−Λ2S2+12μμ+βM0+σ2M022.

Since

(73) $$\begin{array}{c} \mu +\alpha +\gamma +\frac{1}{2}\sigma^{2}{\left(\;\frac{\partial f\left(S^{0},0\right)}{\partial I}\;\right)}^{2}-\beta \frac{\partial f\left(S^{0},0\right)}{\partial I}<0 \end{array}$$

and when *v* > 0 is small enough, it follows that

(74) μ+α+γ+121+vσ2∂fS0,0∂I2−β∂fS0,0∂I<0,−μv+μ+α+γ+121+vσ2∂fS0,0∂I2+βM1<0;

we finally obtain that when *a* > 0 is large enough

(75) $$\begin{array}{c} LV<-1\;\text{a.s. }\forall \left(S,I\right)\in \Gamma \setminus D. \end{array}$$

From Theorem  2.2, given in [10], we know that model (2) has a unique stationary distribution *ξ* such that

(76) PlimT→∞1T∫0TSt,Itdt=∫ΓS,IξdS,I=1.

This completes the proof.

**Remark 25 .**

Comparing Theorem 24 with Theorem  6.2 given in [19], we see that Theorem  6.2 is extended and improved to the general stochastic SIS epidemic model (2).

**Remark 26 .**

Since ${\tilde{R}}_{0}>1$ is equivalent to $\sigma <\bar{\sigma }$, we also have that if $\sigma <\bar{\sigma }$, then model (2) is positive recurrent and has a unique stationary distribution.

Particularly, for some special cases of nonlinear incidence *f*(*S*, *I*), we have the following idiographic results on the stationary distribution as the consequences of Theorem 24.

**Corollary 27 .**

Let *f*(*S*, *I*) = *SI*/*N* (standard incidence). If ${\tilde{R}}_{0}=\left(\beta -\left(1/2\right)\sigma^{2}\right)/\left(\mu +\gamma +\alpha \right)>1$, then model (2) is positive recurrent and has a unique stationary distribution.

**Corollary 28 .**

Let *f*(*S*, *I*) = *h*(*S*)*g*(*I*). Assume that (**H** ^**∗**^) holds and ${\tilde{R}}_{0}=\beta h\left(S^{0}\right)g^{\prime }(0)/\left(\mu +\gamma +\alpha \right)-\sigma^{2}{\left(h\left(S^{0}\right)g^{\prime }\left(0\right)\right)}^{2}/2(\mu +\gamma +\alpha )>1$; then model (2) is positive recurrent and has a unique stationary distribution.

Combining Corollary 6, Theorem 11, Remark 12, Theorem 24, and Remark 26, we can finally establish the following summarization result by using intensity *σ* of stochastic perturbation and basic reproduction number *R*_0_ of deterministic model (1).

**Corollary 29 .**

(a) Let *R*_0_ ≤ 1. Then for any *σ* > 0 the disease in model (2) is extinct with probability one.

(b) Let 1 < *R*_0_ ≤ 2. Then for any $0<\sigma <\bar{\sigma }$ model (2) is permanent in the mean with probability one and has a unique stationary distribution, and for any $\sigma >\bar{\sigma }$ the disease in model (2) is extinct with probability one.

(c) Let *R*_0_ > 2. Then for any $0<\sigma <\bar{\sigma }$ model (2) is permanent in the mean with probability one and has a unique stationary distribution, and for any *σ* > *σ*_1_, where *σ*_1_ is given in (20), the disease in model (2) is extinct with probability one.

## 6. Numerical Simulations

In this section we analyze the stochastic behavior of model (2) by means of the numerical simulations in order to make readers understand our results more better. The numerical simulation method can be found in [19]. Throughout the following numerical simulations, we choose *f*(*S*, *I*) = *SI*/(1 + *ωI*), where *ω* > 0 is a constant. The corresponding discretization system of model (2) is given as follows:

(77) Sk+1=Sk+Λ−βSkIk1+αIk+γIk−μSkΔt+SkIk1+αIkσξkΔt+12σ2ξk2−1Δt,Ik+1=Ik+βSkIk1+αIk−μ+γIkΔt+SkIk1+αIkσξkΔt+12σ2ξk2−1Δt,

where *ξ*_*k*_  (*k* = 1,2,…) are the Gaussian random variables which follow standard normal distribution *N*(0,1).

**Example 1 .**

In model (2) we choose Λ = 2000, *β* = 0.60, *μ* = 11, *γ* = 13, *σ* = 0.075, and *α* = 2.

**Figure 1:**
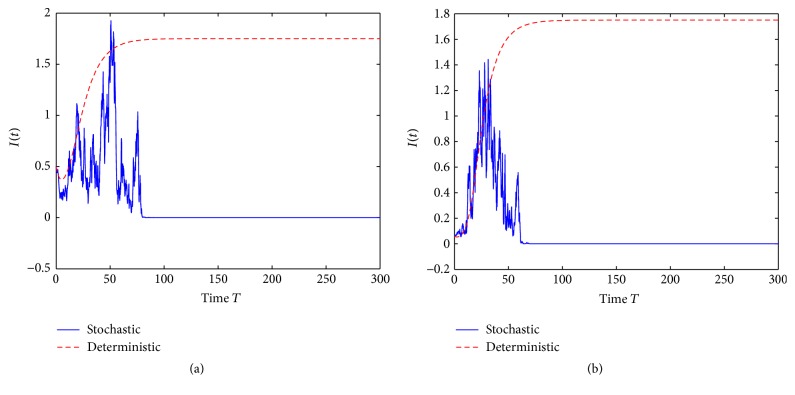
(a) is trajectories of the solution *I*(*t*) with the initial value *I*(0) = 0.5 and (b) with the initial value *I*(0) = 0.06.

By computing we have *R*_0_ = 4.195 > 2, ${\tilde{R}}_{0}=0.6715<1$, *β*/*S* ^0^ − *σ* ^2^ = −0.0023 < 0, and *σ* ^2^ − *β* ^2^/2(*μ* + *γ*) = −0.0019 < 0 which is the case of Remark 9. From the numerical simulations, we see that the disease will die out (see Figure 1). An affirmative answer is given for the open problem proposed in Remark 9.

**Example 2 .**

In model (2), choose Λ = 2000, *β* = 0.9, *μ* = 30, *γ* = 12, and *σ* = 0.09.

**Figure 2:**
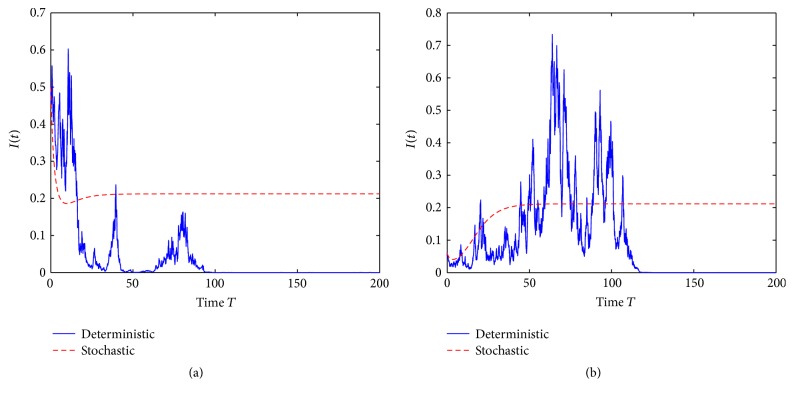
(a) is trajectories of the solution *I*(*t*) with the initial value *I*(0) = 0.5 and (b) with the initial value *I*(0) = 0.06.

By computing we have ${\tilde{R}}_{0}=1$. From the numerical simulations given in Figure 2 we know that the disease will die out. Therefore, an affirmative answer is given for the open problem proposed in Remark 10.

**Example 3 .**

In model (2) choose Λ = 2000, *β* = 0.5, *μ* = 30, *γ* = 20, *σ* = 0.02, and *α* = 2.

**Figure 3:**
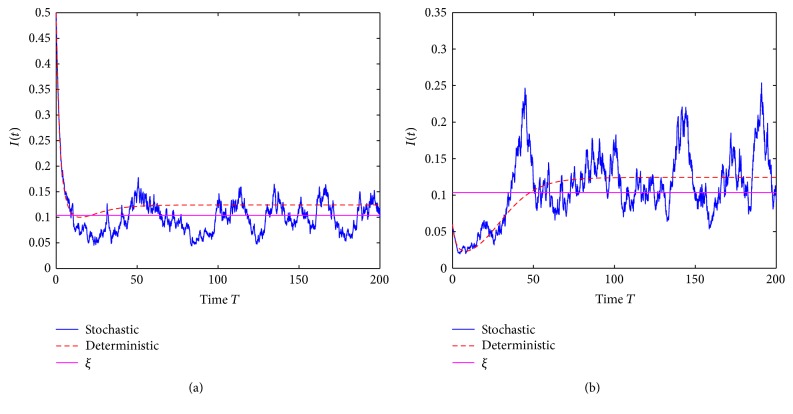
(a) is trajectories of the solution *I*(*t*) with the initial value *I*(0) = 0.5 and (b) with the initial value *I*(0) = 0.06.

We have ${\tilde{R}}_{0}=1.200$, *R*_0_ = 1.2500, and *ξ* = 0.1037. The numerical simulations are found in Figure 3. We can see that solution *I*(*t*) of model (2) oscillates up and down at *ξ*, which further show that the conclusions of Theorems 14 and 18 are true. At the same time, this example also shows that the disease in model (2) is permanent with probability one. Therefore, an affirmative answer is given for the open problems proposed in Remarks 19 and 23.

## 7. Discussion

In this paper we investigated a class of stochastic SIS epidemic models with nonlinear incidence rate, which include the standard incidence, Beddington-DeAngelis incidence, and nonlinear incidence *h*(*S*)*g*(*I*). A series of criteria in the probability mean on the extinction of the disease, the persistence and permanence in the mean of the disease, and the existence of the stationary distribution are established. Furthermore, the numerical examples are carried out to illustrate the proposed open problems in this paper.

It is easily seen that the research given in [6] for the stochastic SIS epidemic model with bilinear incidence is extended to the model with general nonlinear incidence and disease-induced mortality. Particularly, we see that stochastic SIS epidemic model with standard incidence is investigated for the first time.

The researches given in this paper show that stochastic model (2) has more rich dynamical properties than the corresponding deterministic model (1). Particularly, stochastic model (2) has no endemic equilibrium. Thus, this can bring more difficulty for us to investigate model (2), but, on the other hand, this also makes model (2) have more rich researchful subjects than deterministic model (1). We can discuss not only the extinction, persistence, and permanence in the mean of disease in probability, but also the existence and uniqueness of stationary distribution, the asymptotical behaviors of solutions of stochastic model (2) around the equilibrium of deterministic model (1), and so forth.

In addition, we easily see that when intensity *σ* > 0 of the stochastic perturbation, then $R_{0}>{\tilde{R}}_{0}$. This shows that when *R*_0_ > 1 we still can have ${\tilde{R}}_{0}<1$. Therefore, there is a very interesting and important phenomenon; that is, for deterministic model (1) the disease is permanent, but for the corresponding stochastic model (2) the disease is extinct with probability one; see Conclusion (c) of Corollary 29.
